# Development of an Evidence‐Based Yoga Programme for Community‐Dwelling Older Adults: A Mixed‐Methods Pilot Study

**DOI:** 10.1155/nrp/9910769

**Published:** 2026-05-13

**Authors:** Ka-Yan Ko, Helen Yue-Lai Chan, Zoe Ching Man Kwok

**Affiliations:** ^1^ School of Nursing, The Hong Kong Polytechnic University, Hong Kong, China, polyu.edu.hk; ^2^ The Nethersole School of Nursing, Faculty of Medicine, The Chinese University of Hong Kong, Hong Kong, China, cuhk.edu.hk

## Abstract

**Aims:**

To develop an evidence‐based yoga programme and test its feasibility among community‐dwelling older adults.

**Design:**

A mixed‐methods pilot study using a two‐arm randomised controlled trial with qualitative interviews.

**Methods:**

An 8‐week evidence‐based yoga programme was developed for older adults based on a systematic review, guidelines and expert advice. Participants from two community centres were randomly assigned to an intervention group receiving the yoga programme or a control group receiving usual care between January and October 2021. The study’s feasibility was assessed based on rates of recruitment and retention, intervention adherence and adverse events. Acceptability was evaluated by individual semistructured interviews to understand their experience with the programme. Physical and psychological outcomes were measured by the Short Physical Performance Battery and Depression Anxiety Stress Scale‐21, respectively. Preliminary effects of the intervention were examined based on changes in outcomes using a generalised estimation equation, adhering to the intention‐to‐treat principle. Qualitative data were analysed through content analysis.

**Results:**

A total of 74 of 80 participants were eligible and recruited (female: 97.3%; mean age: 67.6 [SD 5.5] years), with an attrition rate of 14.9% (*n* = 11). The mean attendance rate for yoga sessions was 89.9%. Participants found the experience with the programme novel and perceived benefits for pain reduction and improvements in mood and health; however, two‐thirds did not adhere to home practice due to implementation barriers. Exploratory analyses suggested greater improvements in gait speed, flexibility and depressive, anxiety and stress symptoms in the yoga group compared with controls.

**Conclusions:**

This pilot study showed that the yoga programme was feasible and acceptable to community‐dwelling older adults, whereas the adherence to home practice was low, highlighting the need for strategies to enhance motivation.

**Implications:**

This study suggests that the programme can be integrated into primary care to promote healthy ageing. A robust study with a longer follow‐up period is needed to evaluate the intervention effects.

**Trial Registration:** Chinese Clinical Trial Registry: ChiCTR2200054859

**Summary**
• Impact:
◦ Older adults are at risk of physical decline, which likely has negative impacts on psychosocial well‐being.
◦ Our evidence‐based yoga programme is feasible and acceptable, potentially improving physical and psychological health among community‐dwelling older adults.
◦ The programme can be a primary care intervention for promoting intrinsic capacities for healthy ageing.
• Reporting method
◦ Consolidated Standards of Reporting Trials (CONSORT) 2010 statements for randomised pilot and feasibility trials
• Patient or Public Contribution
◦ None

## 1. Introduction

Ageing leads to a gradual decline in physical capacities, negatively affecting balance, muscle strength, flexibility and gait [1–3]. Hence, older adults tend to lead a sedentary lifestyle, resulting in an increased risk of developing sarcopenia and frailty [4]. A decline in physical capacities also affects older adults’ participation in social activities, which may result in depressive symptoms, a sense of alienation and loneliness [5, 6].

The ‘Integrated Care for Older People’ (ICOPE) guidelines introduced by the World Health Organization (WHO) provide guidance on preserving the intrinsic capacity of older adults [7]. The key domains of intrinsic capacities include cognitive, locomotive, vital, psychological, visual and auditory capacities [8]. This comprehensive approach highlights the importance of maintaining both physical and psychological abilities to achieve healthy ageing. Yoga is one of the recommended multimodal exercises in the ICOPE guidelines for preserving both the physical and psychological health of older adults [8].

Over the past 2 decades, growing evidence has shown that practising yoga can improve balance, strength, gait and flexibility among older people [9, 10]. Apart from improving physical functioning, systematic reviews also showed that yoga is effective for enhancing psychological health in older adults [11, 12]. Some studies suggested that the practice of meditation in yoga can improve cognitive performance through autonomic and cytokine‐level alterations [13]. However, previous yoga interventions have predominantly been conducted in Western populations using heterogeneous protocols. Few studies have systematically developed evidence‐based programmes specifically tailored to the needs, culture and physical capacities of Asian community‐dwelling older adults, thus creating a feasibility gap that this pilot study addresses by exploring a locally adapted yoga programme [11].

Recent reviews have identified gaps in knowledge, particularly the limited number of studies pilot‐testing systematically developed yoga interventions based on best available evidence in Asian contexts. In recent years, the ICOPE model has increasingly been used to guide primary health care in Hong Kong, which increasingly recommends multimodal exercises like yoga; necessitating feasibility studies of locally adapted programmes to establish their practicality, cultural acceptability and safety before wider implementation. The aim of this study was to test the feasibility and acceptability of an evidence‐based yoga programme among community‐dwelling older adults. The objectives of the study were to examine (i) the feasibility of the yoga programme in terms of recruitment, retention, adherence and adverse events; (ii) the acceptability of the yoga programme among the participants; and (iii) the preliminary effects of the yoga programme on their physical and psychological health outcomes in terms of balance, gait, strength, flexibility, depression, anxiety and stress. The pilot study findings provide essential feasibility data and preliminary exploratory evidence of potential physical and psychological benefits to inform the evidence‐based yoga programme design of the definitive trial in the future, and potentially guide the integration of group‐based yoga within local primary health promotion strategies for community‐dwelling older adults.

## 2. Methods

### 2.1. Design

We adopted a two‐phase, mixed‐methods design with an explanatory sequential approach, embedding a qualitative study within a two‐arm randomised controlled pilot trial, conducted between January and October 2021. The goal of this design was to use the qualitative data to explain the quantitative findings [14]. In the RCT, participants were randomly assigned to either the control group to receive the usual care or the intervention group to receive an 8‐week evidence‐based yoga programme in addition to the usual care. Quantitative data from the trial were analysed first to examine feasibility outcomes, such as recruitment, retention, adherence and adverse events, and exploratory physical and psychological outcomes. Subsequently, individual semistructured interviews were conducted with participants in the intervention group to understand their experience with the programme and factors influencing adherence. Results were then used to explain and contextualise the quantitative findings, for example, the patterns of attendance and home practice. Study outcomes in physical functioning and psychological outcomes were assessed at baseline and at 8 weeks postallocation for preliminary findings about the potential effects of the interventions. This study was reported according to the Consolidated Standards of Reporting Trials (CONSORT) 2010 statements for randomised pilot and feasibility trials [15]. Besides, this mixed‐methods approach adhered to the Mixed Methods Reporting in Rehabilitation & Health Sciences (MMR‐RHS) standards to ensure transparent and rigorous reporting, and effective integration of quantitative and qualitative components [16].

### 2.2. Setting and Participants

The study was conducted at two government‐funded community centres for older people in different districts in Hong Kong. The criteria for inclusion in the study were those: (i) aged 60 years or above, (ii) mentally competent with a score of 6 or above in the Abbreviated Mental Test Score [17, 18] and (iii) able to understand Cantonese. Excluded were those (i) with serious medical conditions that would interfere with the practice of yoga, e.g., poorly controlled blood pressure, dyspnoea or palpitations; (ii) who had recently undergone major surgery; (iii) who had a severe hearing or visual impairment that could pose a risk to their safety during yoga practice; or (iv) who had participated in other exercises or yoga courses that could interfere with the effects of the current yoga practice. At least 60 participants were required for a pilot study [19]. To account for potential dropouts in the study, the sample size was increased by approximately 20%.

### 2.3. Randomisation and Blinding

Participants were randomly assigned 1:1 to intervention or control groups within each community centre to balance recruitment across sites while facilitating attendance near their homes. An independent research assistant generated centre‐specific random number sequences using a random number table, sealed in opaque envelopes. Eligible participants selected an envelope sequentially to determine allocation, achieving implicit stratification by centre. While participants and instructors could not be blinded due to the intervention’s nature, outcome assessors remained blinded to group assignment to minimise detection bias [20].

### 2.4. Intervention

#### 2.4.1. Usual Care

All of the participants continued to receive their usual care, which included various social and health‐related activities offered by the community centres and medical services provided by hospitals.

#### 2.4.2. An Evidence‐Based Yoga Programme

The yoga programme was developed based on the findings of a systematic review and meta‐analysis [11] and guidelines for yoga interventions [21]. The contents of the programme are shown in Table 1. Hatha yoga was adopted because it has a comprehensive and gentle style. It is generally considered to be beginner‐friendly, making it suitable for older adults to practice [12]. There are four aspects to the practice of yoga: poses (Asanas), breathing control (Pranayamas), meditation (Dhyana) and relaxation (Savasana) [22]. This study adhered to the Checklist Standardising the Reporting of Interventions for Yoga (CLARIFY) 2021 guidelines for the yoga intervention reporting [23]. The 15 yoga poses were introduced one by one to facilitate learning. The participants were allowed to adapt the poses and to use a chair for support, based on their own ability and health conditions. The programme was led by two certified yoga instructors with experience in teaching older adults. It included 16 one‐hour face‐to‐face group sessions, twice a week for eight weeks, which was informed by subgroup analyses from the systematic review, which showed this range associated with optimal improvements in physical and psychological outcomes among older adults. Each group included 10–15 participants, and the sessions were conducted in the community centres. The yoga sessions were scheduled at different times from the control group activities at each centre to minimise contamination between groups. Participants in the intervention group were also advised not to share the intervention materials during the study period. Additionally, participants were encouraged to practice at home for 20 min twice a week, a dosage that is supported by recent evidence showing that such low‐frequency home practice combined with regular yoga classes enhances both physical and cognitive functions in older adults [24, 25]. To support their home practice, an information booklet with photographs of the poses was provided after the first session. Verbal instructions were given to yoga participants not to share the materials or practice information with control group peers to minimise contamination.

**TABLE 1:** List of practices in the yoga programme.

Order	Yoga components	Time	Benefits of postures	Chair support
1	Starting Mindfulness grounding	2 min	Mind‐body connection	√

2	Warm‐up Seated mountain pose Neck stretching Seated side stretch Seated simple twist Seated cat‐cow pose	3 min	Stretching, increases flexibility and strengthens the lower back	√

3	Main poses Chair down‐dog pose High lunge Chair pose Tree pose Triangle pose Warrior II pose Chair pigeon pose Chair forward bend Bridge pose Butterfly pose	30 min	Stretches the shoulders, back, knees, hips and hamstrings. Improves balance, increases flexibility and strengthens the leg muscles.	√
4	Meditation, relaxation with breathing control Abdominal breathing or Bhramari Pranayama (bee breathing) Savasana (body‐mind connection)	5 min	Relaxes and brings harmony between the body, mind and spirit.	

#### 2.4.3. Intervention Fidelity

The content validity of the intervention protocol was established by two well‐trained yoga instructors, certified by the Yoga Alliance of the USA (with RYT 500 qualifications) and have more than 5 years of experience in teaching older adults’ yoga. During the intervention, researchers and instructors held regular meetings to review participant performance and protocol fidelity. The first author conducted weekly site visits to monitor session delivery, instructor adherence to the yoga protocol and overall programme fidelity. During each visit, a structured protocol adherence checklist was completed, documenting the date, adherence percentage and any deviations observed. Session attendance was systematically recorded by community centre staff, with absent participants contacted to document reasons for nonattendance. Phone calls and messages served as reminders for both yoga sessions and scheduled assessments. Additionally, participants were required to log the date, time and duration of their home yoga practice. Their logbooks were reviewed weekly during site visits to monitor adherence, track progress and address any concerns.

### 2.5. Study Outcomes

#### 2.5.1. Feasibility

The feasibility of the intervention was assessed using Avery’s traffic light progression criteria [26], which evaluate recruitment rate, recruitment duration, adherence, attrition and adverse events. Progression thresholds were prespecified across three phases: green, yellow and red lights, with each representing different levels of feasibility and readiness for the intervention to be implemented [26]. An intervention was assigned a green light when it was considered feasible and ready for a definitive trial, with the recruitment ≥ 50%, adherence ≥ 80%, attrition rate ≤ 20% and no adverse events noted. A yellow light indicated that a cautious approach needs to be taken (recruitment 30%–49%, adherence 60%–79%, attrition 21%–30%) and that modifications might be required of the intervention. A red light (recruitment < 30%, adherence < 60%, attrition > 30% or serious adverse events noted) denotes that the intervention might be unfeasible unless significant modification is made [27].

#### 2.5.2. Acceptability

The acceptability of the intervention was assessed by class attendance with a target ≥ 80% and adherence rates of ≥ 65% to home practice, and these thresholds aligned with benchmarks from [9]. The participants’ experiences with the intervention and their perceptions of it were explored through individual semistructured interviews after the completion of the intervention.

#### 2.5.3. Physical Outcomes

The Short Physical Performance Battery (SPPB) was used to assess the participants’ physical functions in terms of balance, flexibility, muscle strength and gait. Their mobility was assessed by their gait speed during a four‐m walk, their lower limb strength during the completion of five chair‐stands, and their balance in three separate tests [28]. Each component contributed to a composite score ranging from 0 to 12, with higher scores indicating better physical performance. In this study, to enhance the sensitivity of the assessment, gait was also measured in terms of the time required to complete the walking test (in seconds), rather than solely according to categorical scores. This approach provided a more precise evaluation of changes in lower limb performance than that reflected by categorical scores alone. SPPB is a simple test, with a high validity and reliability of 0.87 (95% CI 0.77–0.96). It has been widely used for evaluating the mobility and physical functions of older adults [28]. In addition, flexibility was assessed using the sit‐reach test, which is widely used for testing the flexibility of the lower back and hamstrings [29]. The physical outcome measures were collected by a research assistant blinded to participants’ group allocations in a dedicated room at the community centre.

#### 2.5.4. Psychological Outcomes

The Depression Anxiety Stress Scale‐21 (DASS‐21) was used to measure three emotional states: depression, anxiety and stress. It consists of 21 items, with each item scored from zero (did not apply to me at all) to three (applied to me very much). A higher score represents a higher severity of emotional distress [30]. It is a valid and reliable tool that is used to assess the psychological health of adults. The Chinese version, which has a reliability of 0.912, was used in the current study [31]. The DASS‐21 assessments were also conducted in a dedicated room at the community centre by a research assistant blinded to participants′ group allocations.

### 2.6. Safety Monitoring and Adverse Events

The safety of the yoga practice was monitored by the yoga instructors and the staff of the community centres during the yoga session. Adverse events were defined as any undesirable symptoms or responses occurring during sessions or home practice (e.g., pain, dizziness, falls). Community centre staff maintained emergency contacts and comprehensive insurance coverage for all participants, arranging immediate medical treatment and family notification as needed for any adverse events. Yoga instructors routinely inquired about home practice incidents at the start of each session.

### 2.7. Ethical Considerations

Ethical approval was obtained from the Joint Chinese University of Hong Kong New Territories East Cluster Research Ethics Committee (Joint CUHK‐NTEC CREC). The first author explained the nature and purpose of the study to potential participants. Written informed consent was obtained from eligible persons before the study. Participation in the study was voluntary. Participants had the right to withdraw from the study at any time.

### 2.8. Data Collection

The study was promoted through advertisements in newsletters and leaflets in the community centres. Interested persons enrolled at the centres and underwent screening for eligibility. Sociodemographic information and baseline assessments were collected from the participants. During the intervention, a booklet was distributed to each yoga participant so that they could self‐record the frequency and duration of their yoga practice at home, as well as any adverse events related to their practice of yoga.

### 2.9. Data Analysis

A statistical analysis was conducted using IBM SPSS Statistics Version 28. Descriptive statistics were used to summarise the participants’ characteristics and study outcomes. The physical and psychological outcomes of the two groups were compared using generalised estimation equation (GEE) models. The analyses followed the intention‐to‐treat principle. All interviews were audio‐recorded and transcribed verbatim for data analysis. The qualitative data were analysed using qualitative content analysis [32]. The researchers read through the transcripts several times to familiarise themselves with the content. The first author took the lead in extracting the meaning units and comparing them. Similar units were grouped into categories. The research team then reviewed, discussed and refined the categories through email communications and meetings.

## 3. Results

### 3.1. Recruitment of Participants

**FIGURE 1:**
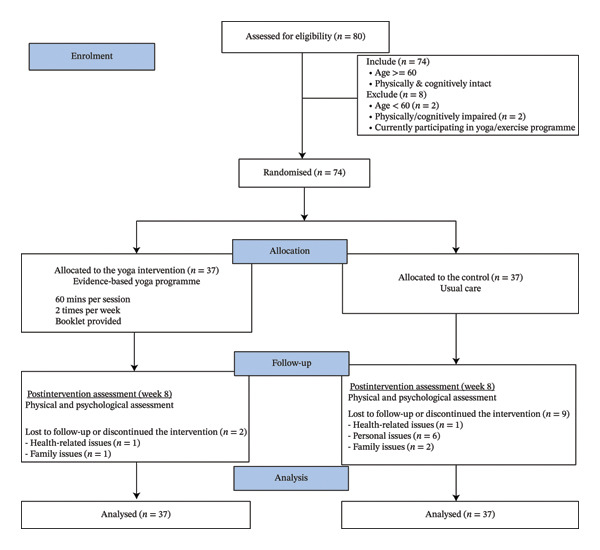
CONSORT flow diagram.

A total of 80 older adults were assessed for eligibility. Six participants failed to meet the inclusion criteria and were excluded from the study: two were less than 60 years old, two had impaired cognitive or physical functions, and two were already participating in a similar yoga programme. The eligibility rate was 92.5%. The recruitment process was completed in 2 months. The 74 participants were randomly assigned to either the control group or the intervention group, with 37 per group. Figure 1 shows the CONSORT diagram.

### 3.2. Characteristics of the Participants

Participants’ mean age was 67.6 (SD 5.5) years, ranging from 60 to 92. The participants in the intervention group were significantly older than those in the control group (69.9 ± 5.9 vs 65.1 ± 3.5, *p* = 0.001). Approximately two‐thirds of them were living with family members, and about the same proportion had a secondary education or above. The participants (90.5%) generally had no experience with yoga. Their sociodemographic data are shown in Table 2.

**TABLE 2:** Sociodemographic characteristics.

	**All (*N* = 74)**	**Control group (*n* = 37)**	**Intervention group (*n* = 37)**

Age, mean (SD)	67.6 (5.5)	65.1 (3.5)	69.9 (5.9)
Gender			
Female	72 (97.3%)	35 (94.6%)	37 (100%)
Male	2 (2.7%)	2 (5.4%)	0 (0%)
Marital status			
Single/widowed/divorced	40 (54.1%)	17 (45.9%)	23 (62.2%)
Married	34 (45.9%)	20 (54.1%)	14 (37.8%)
Education level			
No formal education	8 (10.8%)	6 (16.2%)	2 (5.4%)
Primary	22 (29.7%)	13 (35.1%)	9 (24.3%)
Secondary	33 (44.6%)	15 (40.5%)	18 (48.6%)
Tertiary or above	11 (14.9%)	3 (8.1%)	8 (21.6%)
Living status			
Living with family	46 (62.2%)	25 (67.6%)	21 (56.8%)
Living alone	28 (37.8%)	12 (32.4%)	16 (43.2%)
Social security allowance			
No	32 (43.2%)	20 (54.1%)	12 (32.4%)
Yes	42 (56.8%)	17 (45.9%)	25 (67.6%)
Current working status			
Unemployed	68 (91.9%)	32 (86.5%)	36 (97.3%)
Employed	6 (8.1%)	5 (13.5%)	1 (2.7%)
Katz ADL	6 (0.0)	6 (0.0)	6 (0.0)

*Note:* Data are presented as numbers (frequency), unless specified.

Abbreviations: ADL, activities of daily living; AMT, abbreviated mental test; SD, standard deviations.

### 3.3. Acceptability

#### 3.3.1. Class Attendance

The mean attendance in the yoga classes was 89.9%, with 74% of the participants attending at least 70% of all yoga sessions (more than 12 out of 16 sessions in total). Reasons for being absent from the yoga classes included attending medical follow‐up appointments, being on sick leave and family issues. The attrition rate at T1 was 14.9%.

#### 3.3.2. Intervention Adherence

Only 13 (37.9%) participants performed self‐directed practice at home twice per week. Eleven (29.7%) participants engaged in self‐practice at home once a week. The remaining participants never practised at home or did not have clear documentation of their self‐practice.

#### 3.3.3. Experience With the Intervention

The majority of the participants experienced yoga for the first time. They generally found it a new learning experience. Two main categories emerged from the qualitative interviews regarding participants’ experiences with the yoga intervention: perceived benefits and barriers to practice. These findings explained the discrepancy between high‐class attendance (89.9%) and low home practice adherence (37.9%). Table 3 presents a joint display mapping qualitative themes to quantitative feasibility metrics, showing how novel learning experience and social interaction enjoyment explained the high attendance rate for yoga classes, while the environmental and individual barriers elucidated the low home practice adherence.

**TABLE 3:** Joint display for representing mixed‐method findings.

Findings	Quantitative findings	Qualitative themes/quotes
Attendance	89.9% (Yoga sessions attended; 74% attended ≥ 70% of sessions)	‘Novel experience, group enjoyment’ ‘I feel happy and excited to have yoga sessions with all the others’ (P008) ‘I preferred to practice yoga with other groupmates’ (P005)

Home practice adherence	37.9% (2 times/week); 29.7% (1 time/week); 32.4% (none/unclear)	Environmental barriers ‘Don’t have sufficient space at home to place a yoga mat’ (P030) Individual barriers ‘Too lazy… no motivation’ (P005); ‘Feel lonely practicing alone’ (P016)

Physical outcomes	‐ Gait speed: ↑ (*p* < 0.001) ‐ Flexibility: ↑ (*p* < 0.001) ‐ SPPB total: ↑ (*p* < 0.001) ‐ Balance/strength: No significant change	Perceived physical benefits: ‘Less knee pain… now can bend my knee’ (P013) ‘Improved flexibility and gait’ (P003) ‘Learned how to sit or get up safely…’ (P003)

Psychological outcomes	‐ DASS‐21 total: ↓ (*p* < 0.001) ‐ Depression: ↓ (*p* < 0.001) ‐ Anxiety: ↓ (*p* < 0.001) ‐ Stress: ↓ (*p* < 0.001)	Perceived psychological benefits: ‘Improved sleep quality… more comfortable mood’ (P028) ‘Feel energetic and revitalized’ (P008)

##### 3.3.3.1. Perceived Benefits

There were two subcategories of the participants’ perceptions of the benefits of practising yoga. These included both physical benefits and psychological benefits.

• Perceived physical benefits. Many of the participants noted improvements in their physical condition. They believed that yoga helped to alleviate discomfort and pain in their back, knees, shoulders and joints. Some participants said that after completing the yoga practice, their sitting posture had improved.

“*I think yoga poses helped me to stretch my muscles and tendons, which improved my flexibility and gait. Now I have learned how to sit down and get up from the floor safely and appropriately after the yoga instructor taught me. Also, I think the frequent practice of yoga might help to improve my health.*” (P003)

“*My right knee was very painful due to degeneration, and I had a medical follow-up at the orthopaedic outpatient clinic. Now I feel less pain and I’m able to bend my right knee; I can even sit on the floor…*.” (P013)

• Perceived psychological benefits. Many participants found that yoga made them feel energetic and revitalised, and also gave them a sense of satisfaction and pleasure. They stated that they experienced improved mood, reduced instances of insomnia, enhanced sleep quality and heightened self‐confidence. Some also enjoyed the group interactions during the intervention sessions, as these enhanced their social connections.

*“I have never practiced yoga before and had no idea about yoga. I feel happy and excited to have yoga sessions with all the others, and I like the slow pace and poses of yoga.”* (P008)

*“I enjoyed practicing yoga. It improved my sleep quality. I felt more comfortable with my mood and body after practicing yoga.”* (P028)

##### 3.3.3.2. Perceived Barriers to Practising Yoga

The barriers to practising yoga were divided into two subcategories: environmental factors and individual factors.

• Environmental Factors. Some participants seldom or never engaged in self‐practice at home. They identified some environmental barriers to practising yoga at home, including insufficient space and facilities.

*“I don’t have sufficient space at home to place a yoga mat on the floor, and … I don’t know where to get the right kind of mat for self-practice.”* (P030)

• Individual factors. The participants lacked the confidence to practice yoga on their own due to safety concerns, despite having been given a yoga self‐practice booklet. Some participants thought that they would not be able to remember the yoga poses due to poor memory. Other reasons deterring them from practising yoga at home included laziness and time constraints. Furthermore, participants preferred group activities to practising yoga alone at home.

*“I know I am too lazy to practice yoga at home, and I don’t have the motivation to do so. I preferred to practice yoga with other groupmates during the yoga sessions every week. I think two yoga lessons per week are enough for me, and I don’t need to practice it myself at home.”* (P005)

*“I would feel lonely practicing yoga by myself at home. I preferred to take part in group activities.”* (P016)

### 3.4. Preliminary Effects of the Yoga Programme

#### 3.4.1. Physical Outcomes

Table 4 shows the physical outcomes, in terms of balance, gait, strength and flexibility, reported at T0 and T1. The GEE results showed a significant group–time interaction effect in the total SPPB score (*β* = 2.712; 95% CI = 2.129–3.295; *p* < 0.001). The intervention group demonstrated a significantly greater improvement in the flexibility required for the sit‐reach test (*β* = 5.483; 95% CI = 4.128–6.838; *p* < 0.001). There were no significant group differences in the subscale scores of balance, gait and strength in the SPPB. However, there was a significant group–time interaction effect in the time required to complete the 4‐m walk test (*β* = −1.709, 95% CI = −2.350–−1.068, *p* < 0.001), showing that the intervention group had a significantly faster gait speed than the control group.

**TABLE 4:** Generalised estimating equation (GEE) for physical outcomes.

Outcome variables	Time	Mean ± SD		Group effect		Time effect		Group ∗ time effect	
		Yoga group	Control group	*β* (95% CI)	*p*	*β* (95% CI)	*p*	*β* (95% CI)	*p*
SPPB—total score	T0	8.22 ± 1.77	9.16 ± 1.61	−0.946 (−1.705, −0.187)	0.015	0.173 (−0.168, 0.515)	0.321	2.712 (2.129, 3.295)	< 0.001
	T1	11.17 ± 1.25	9.32 ± 1.54						

Balance	T0	3.89 ± 0.32	3.58 ± 0.62	0.053 (−0.159, 0.265)	0.624	−0.636 (−1.100, −0.173)	0.007	0.053 (−0.579, 0.027)	0.869
	T1	3.44 ± 0.75	3.59 ± 0.67						

Gait	T0	2.14 ± 0.79	2.68 ± 0.60	0.242 (−0.360, 0.844)	0.430	−0.459 (−1.128, 0.210)	0.179	−0.459 (−1.128, 0.210)	0.179
	T1	3.56 ± 0.62	2.86 ± 0.56						

Strength	T0	2.62 ± 1.01	3.00 ± 0.89	0.333 (−0.448, 1.114)	0.403	1.194 (0.854, 1.534)	< 0.001	−0.527 (−1.117, 0.062)	0.079
	T1	2.68 ± 0.64	2.77 ± 0.97						

Time required for 4‐m walk test (sec)	T0	6.75 ± 1.59	5.77 ± 1.26	0.977 (0.332, 1.623)	0.003	−0.579 (−0.958, −0.199)	0.003	−1.709 (−2.350, −1.068)	< 0.001
	T1	4.44 ± 1.01	5.19 ± 0.88						

Sit‐reach test (cm)	T0	11.95 ± 4.36	11.43 ± 3.58	0.519 (−1.34, 2.377)	0.584	0.242 (−0.448, 0.932)	0.491	5.483 (4.128, 6.838)	< 0.001
	T1	17.46 ± 3.84	11.97 ± 3.94						

*Note:* T0, baseline; T1, 8‐week follow‐up.

#### 3.4.2. Psychological Outcomes

Table 5 shows the psychological outcomes reported at T0 and T1. The GEE results revealed a significant group–time interaction effect in the total DASS‐21 scale (*β* = −7.621; 95% CI = −4.479–−10.764; *p* < 0.001), and in all subscales, including depressive symptoms (*β* = −2.564; 95% CI = −3.855–−1.273; *p* < 0.001), anxiety (*β* = −1.915; 95% CI = −2.797–−1.033; *p* < 0.001) and stress (*β* = −3.135; 95% CI = −4.596–−1.675; *p* < 0.001). The intervention group generally reported a significantly greater reduction in all areas of psychological distress than did the control group.

**TABLE 5:** Generalised estimating equation (GEE) for psychological outcomes.

Outcome variables	Time	Mean ± SD		Group effect		Time effect		Group ∗ time effect	
		Yoga group	Control group	*β* (95% CI)	*p*	*β* (95% CI)	*p*	*β* (95% CI)	*p*
DASS—total score	T0	10.68 ± 10.30	5.73 ± 6.10	4.946 (1.14, 8.75)	0.011	−0.017 (−2.075, 2.041)	0.987	−7.621 (−10.764, −4.479)	< 0.001
	T1	2.79 ± 3.21	5.64 ± 5.30						

Depression	T0	3.19 ± 3.73	2.46 ± 2.92	0.730 (−0.777, 2.236)	0.342	0.124 (−0.813, 1.060)	0.796	−2.564 (−3.855, −1.273)	< 0.001
	T1	0.68 ± 0.98	2.57 ± 2.54						

Anxiety	T0	3.11 ± 3.43	0.68 ± 1.08	2.432 (1.289, 3.576)	< 0.001	−0.237 (−0.588, 0.115)	0.187	−1.915 (−2.797, −1.033)	< 0.001
	T1	0.85 ± 1.31	0.39 ± 0.69						

Stress	T0	4.38 ± 4.26	2.59 ± 2.76	1.784 (0.170, 3.397)	0.030	0.094 (−0.950, 1.138)	0.860	−3.135 (−4.596, −1.675)	< 0.001
	T1	1.26 ± 1.88	2.68 ± 2.64						

*Note:* T0, baseline; T1, 8‐week follow‐up.

### 3.5. Adverse Events

No serious adverse events were reported in relation to the practice of yoga. Two participants complained of mild muscle pain during the fifth and sixth weeks of the yoga sessions, but their condition subsided afterwards. Yoga instructors explained that this was common for people who seldom or never performed regular physical exercise.

## 4. Discussion

This paper reports on a mixed‐methods study conducted to evaluate the feasibility and acceptability of an evidence‐based 8‐week yoga programme specifically designed for older adults. The qualitative data served to contextualise the quantitative findings and offer a more comprehensive overview of the feasibility and acceptability of the evidence‐based yoga programme [33]. The results demonstrated its feasibility through a high recruitment rate and class attendance rate, and the absence of adverse events. However, the overwhelming female predominance (97.3%) in the sample reflects established gender barriers to yoga participation among older men, for example, gender norms, perceptions of yoga as ‘feminine’, and male preferences for alternative activities [34, 35]. This pilot study was not powered for effectiveness testing, and therefore, the between‐group differences in physical and psychological outcomes showed that exploratory data analyses should be interpreted with caution due to small sample size, significant between‐group differences in age and absence of attention control.

Most feasibility outcomes reached the green light stage, indicating that they met the recommended progression criteria, as based on Avery’s traffic light progression criteria [27]. However, only one‐third of the participants had adhered to home practice of yoga twice per week, suggesting that the predefined acceptability threshold ≥ 65% for this intervention component was not met [9]. The result of class attendance in this study was similar to that in recent systematic reviews by Sivaramakrishnan et al. [12] and Tulloch et al. [36], which found the attendance rates ranged from 53% to 100%. Qualitative interviews explained the feasibility discrepancy between high‐class attendance and low home practice through environmental barriers, such as insufficient space or facilities and individual factors like safety concerns, low motivation and preference for group practice. The intervention demonstrated conditional progression, indicating that the group‐based yoga delivery was feasible and acceptable; however, the home‐based yoga practice required redesign, such as the integration of digital support, equipment provision or instructor check‐ins to enhance adherence. However, the attrition rate in this study was higher than that reported in previous studies, which ranged from 7.9% to 10.8% [9, 37, 38]. Notably, most dropouts occurred in the control group, suggesting that attention control activities may be helpful in future trials to enhance retention and minimise bias [39].

Our exploratory findings are consistent with those of other studies in that there were greater improvements in physical functioning measured in terms of flexibility and gait among participants in the intervention group than those in the control group [10, 38, 40–42]. These differences might be because various yoga poses in our programme involve stretching, which promotes flexibility and reduces stiffness [43]. Flexibility also assists in maintaining balance and gait, which are crucial for preventing falls [40, 43]. However, given the study’s limitations, these findings cannot be used for hypothesis testing. On the other hand, experience from this pilot study revealed the need to examine the appropriate method for evaluating gait performance. A significant group–time interaction was observed in gait speed, as measured by the time required to complete the 4‐m walk test, but not in the gait subscale of the SPPB. This inconsistent result might stem from differences in the sensitivity of these two measurement methods. Possibly, measuring exact times is likely more precise than relying solely on a categorical score to reflect the physical abilities of participants, which is supported by a few studies [44, 45].

However, nonsignificant balance and strength changes were found, which likely reflected the ceiling effects in this relatively healthy cohort of participants [44]. Moreover, the 8‐week follow‐up period was relatively short because building muscle strength in older age requires a longer period of practice [46]. The qualitative findings further complemented the quantitative findings by showing the benefits of practising yoga, including improvements in sleep quality and pain alleviation, which were not measured quantitatively. The alleviation of pain might have occurred because increased flexibility reduces muscle tension and enables older people to walk with a proper gait [47, 48].

This study also found that the intervention group reported a reduction in psychological distress after the yoga intervention. This preliminary finding is consistent with previous research and systematic reviews [12, 38]. One possible reason is that yoga’s meditation and breathing techniques reduce blood pressure, heart rate and cytokine levels, regulating the autonomic nervous system and lowering cortisol to promote relaxation [49, 50]. The long‐term practice of yoga and meditation might modulate the activity in regions of the brain involved in processing emotions, which could enhance the ability to regulate emotions and result in higher levels of self‐compassion [51–53]. On the other hand, a systematic review found that physical activity in a group‐based format is effective for reducing depressive symptoms among older adults [54]. The exploratory psychological improvements across DASS‐21 subscales may be explained by the social interactions during the group‐based activities.

### 4.1. Study Limitations

This study has several limitations. First, the marked female predominance (97.3%) limited the generalisability to males, consistent with yoga trials where female enrolment predominates due to the ‘feminine’ perceptions of yoga, and male preferences for alternative activities [55]. This introduced selection bias that may substantially limit applicability to male populations. Second, the randomisation was conducted at the individual level; however, this may have introduced a risk of contamination, as intervention and control participants shared community facilities. Future trials should consider cluster randomisation by centre to minimise this risk. The small sample size of the current study precluded definitive efficacy conclusions for yoga’s effects on physical or psychological outcomes, demonstrating a power limitation typical for feasibility studies. Also, the psychological outcomes and home practice logbooks are based on self‐reports, which are susceptible to social desirability and might induce bias. Furthermore, considering the primary aim of the current study was feasibility and acceptability testing, the attention control group was absent, potentially inflate preliminary effects observed [56].

### 4.2. Implications

Yoga has been recommended by the WHO as a multimodal exercise for promoting the intrinsic capacity to achieve healthy ageing. The findings of this study have several implications for practice and research. First, a group‐based format for practising yoga should be promoted in primary care because it bolsters motivation for continued practice. Second, given the gender bias in participation in yoga, groups exclusively for males should be formed to create a more comfortable environment for male participation [56]. Third, a full‐scale, robust study with an attention control group and a longer follow‐up assessment period should be conducted to evaluate the effects of the evidence‐based yoga programme on the physical and psychosocial health of older adults. Fourth, additional outcome measures such as the 6‐min walk test (6MWT) or the Timed Up and Go (TUG) test can be used in future studies to provide a more precise assessment of gait and strength outcomes, respectively, rather than relying solely on a scoring system.

## 5. Conclusion

This study found that an evidence‐based yoga programme is feasible and acceptable for older adults in primary care in community settings. The exploratory findings suggested potential directions for physical and psychological benefits, and the feasibility and acceptability data provide essential groundwork for a full‐scale robust study to determine the effects of yoga on community‐dwelling older adults.

## Author Contributions

Helen Yue‐Lai Chan contributed to the supervision of the study and guided the study design, methodology and manuscript revision. Ka‐Yan Ko contributed to the study design, implementation, drafting, writing and revision of the manuscript. Zoe Ching Man Kwok contributed comments and assisted with the study implementation process.

## Funding

This study received no external funding.

## Conflicts of Interest

The authors declare no conflicts of interest.

## Data Availability

The data that support the findings of this study are available from the corresponding author upon reasonable request.
